# Atypical presentation of lateral periodontal cyst associated with impacted teeth: two case reports

**DOI:** 10.1186/s12903-021-01539-7

**Published:** 2021-04-07

**Authors:** S. Buchholzer, Fabien Bornert, D. Di Donna, T. Lombardi

**Affiliations:** 1 Oral Medicine and Maxillofacial Pathology Unit, Division of Oral and Maxillofacial Surgery, Department of Surgery, Geneva University Hospitals, Rue Gabrielle-Perret-Gentil 4, 1205 Geneva, Switzerland; 2 Private Practice, Geneva, Switzerland

**Keywords:** Lateral periodontal cyst, Odontogenic cyst, Jaw, Case report

## Abstract

**Background:**

Lateral periodontal cyst (LPC) is an uncommon form of developmental odontogenic cyst. LPC can be suspected when there is a round, well-circumscribed radiolucency, usually of small diameter, along the lateral surface of vital erupted teeth, predominantly in the mandibular premolar region. Histopathological analysis allows LPC to be diagnosed based on its characteristic features such as a thin cuboidal to stratified squamous non-keratinizing epithelium containing epithelial plaques and glycogen-rich clear cells.

The aim of this article was to report two cases of atypical LPC associated either with an impacted lower left canine (tooth #33) or with a lower right third molar (tooth #48).

**Case presentation:**

*Case 1*: A 56-year-old man was referred to us for an oro-dental assessment. Panoramic radiography revealed an impacted lower left permanent canine (tooth #33) with well-defined radiolucency on its upper cervical margin. A CT scan revealed a pericoronal radiolucency of 5 mm at its widest diameter around the impacted tooth #33. The pericoronal tissue was removed and sent for histopathological examination. The results revealed a lateral periodontal cyst. Satisfactory postoperative healing was achieved at the site. Follow-up at 12 months indicated no recurrence of the lesion.

*Case 2*: A 54-year-old woman consulted with the main issue being pain on the lower right side of the face. Intra-oral examination revealed a vestibular swelling involving the region of the second molar (tooth #47), with obliteration of buccal sulcus. Pocket depth was determined to be 9 mm at the distal of #47. A diagnosis of gingival abscess resulting from chronic periodontitis was made. Panoramic radiography revealed a radiolucent cystic lesion associated with an impacted horizontal lower right third molar (tooth #48), suggestive of a dentigerous cyst. X-rays also revealed alveolar bone resorption on the molar (tooth #47). The cyst was removed along with the third molar and submitted for histopathological diagnosis. The diagnosis was LPC. Follow-up at 18 months indicated no recurrence of the lesion.

**Conclusion:**

These cases represent atypical presentations of LPC. They provide examples of the differential diagnosis of pericoronal radiolucencies involving an impacted tooth and our observations provide insights regarding the pathogenesis of LPC.

## Background

Lateral periodontal cyst (LPC) is a relatively rare benign intra-osseous epithelial developmental odontogenic cyst that represents 0.7% [1] to 1.5% [2] of all cysts of the jawbone. LPC was first described by Standish and Shafer in 1958 [3] as a benign and indolent lesion with minimal growth potential and a low rate of recurrence.

LPC is usually diagnosed in individuals who are between 40 and 70 years of age, predominantly in the fifth and sixth decades [4]. It is thought to affect both genders equally, although some studies have reported a slight male predominance of 1.3:1 [1, 3].

LPCs are usually symptomless and discovered fortuitously on routine radiological examination. In some cases, LPC can also be revealed by a swelling on the vestibular side of the alveolar process in relation to their growth, which expands the overlying bone [1]. Erosion of the cortical plate can occur, thus involving both bone and gingival tissues, leading to a bluish gingival swelling that can result in local pain [3].

The typical radiological presentation of LPC is a well-defined round, ovoid, teardrop shape or inverted pear radiolucency measuring less than a centimeter in diameter and surrounded by a sclerotic margin [1, 3]. LPCs are usually located between the apex and the cervical margin of an erupted vital tooth, in close proximity to its periodontal space [4]. Divergence of the dental roots is not uncommon [1], although root resorption occurs very rarely [5].

The mandibular premolar region is the most common site of LPC formation, followed by the canine-incisal mandibular area and the anterior maxillary region. In rare cases, an LPC can occur around a molar. [1–4]

Botryoid odontogenic cyst (BOC) is a variant of the LPC [6], and it was first described by Weathers and Waldron in 1973 [3]. BOC usually presents as a more extensive multilocular radiolucency, although -like LPC- it can also present as a unilocular lesion. BOCs are more frequently symptomatic, inducing swelling and pain [1]. The BOC variant can be clearly distinguished from LPC by histological analysis [3] and it has a higher recurrence rate compared to LPC [1].

LPC was added to the 2nd edition of the World Health Organization (WHO) classification in 1992 as an odontogenic developmental epithelial cyst of the jawbone, and reference was made to its multilocular BOC variant. However, the third edition of the WHO classification in 2005 excluded odontogenic cysts. The more recent WHO classification published in 2017 re-included cysts, and BOC has been added as a polycystic variant of LPC [7, 8].

LPCs are histologically characterized by a unicystic cavity lined by one to five thin layers of a cuboidal to stratified non-keratinizing squamous epithelial cells, supported by a connective tissue wall usually devoid of inflammatory cells that can also contain clear cells with epithelial rests of Malassez. The two main characteristic features of LPCs are: epithelium thickenings or plaques and clear cells rich in glycogen, which can be seen either in the epithelium plaques or on the superficial layers of the epithelium [1, 5].

The origin of LPCs remains controversial, with extensive debate in the literature regarding the various etiopathological hypotheses [4].

Treatment of LPC generally involves complete enucleation. The risk of recurrence of LPC is estimated to be 3% to 4% and it usually occurs several years later [3].

The aim of this article was to report two unusual cases of LPC associated with impacted teeth, emphasizing the differential diagnosis and providing new insights regarding the etiopathogenesis.

## Case presentation

### Case 1

A 56-year-old man was referred to the Oral Medicine and Maxillofacial Pathology Unit for an oro-dental assessment before chemo-radiotherapy treatment for a squamous cell carcinoma of the right vocal cord classified as T4a N1 M0. The patient had no oro-dental symptoms.

The dental examination revealed partially edentulous upper and lower jaws and dental caries on teeth #34 & #35.

**Fig. 1:**
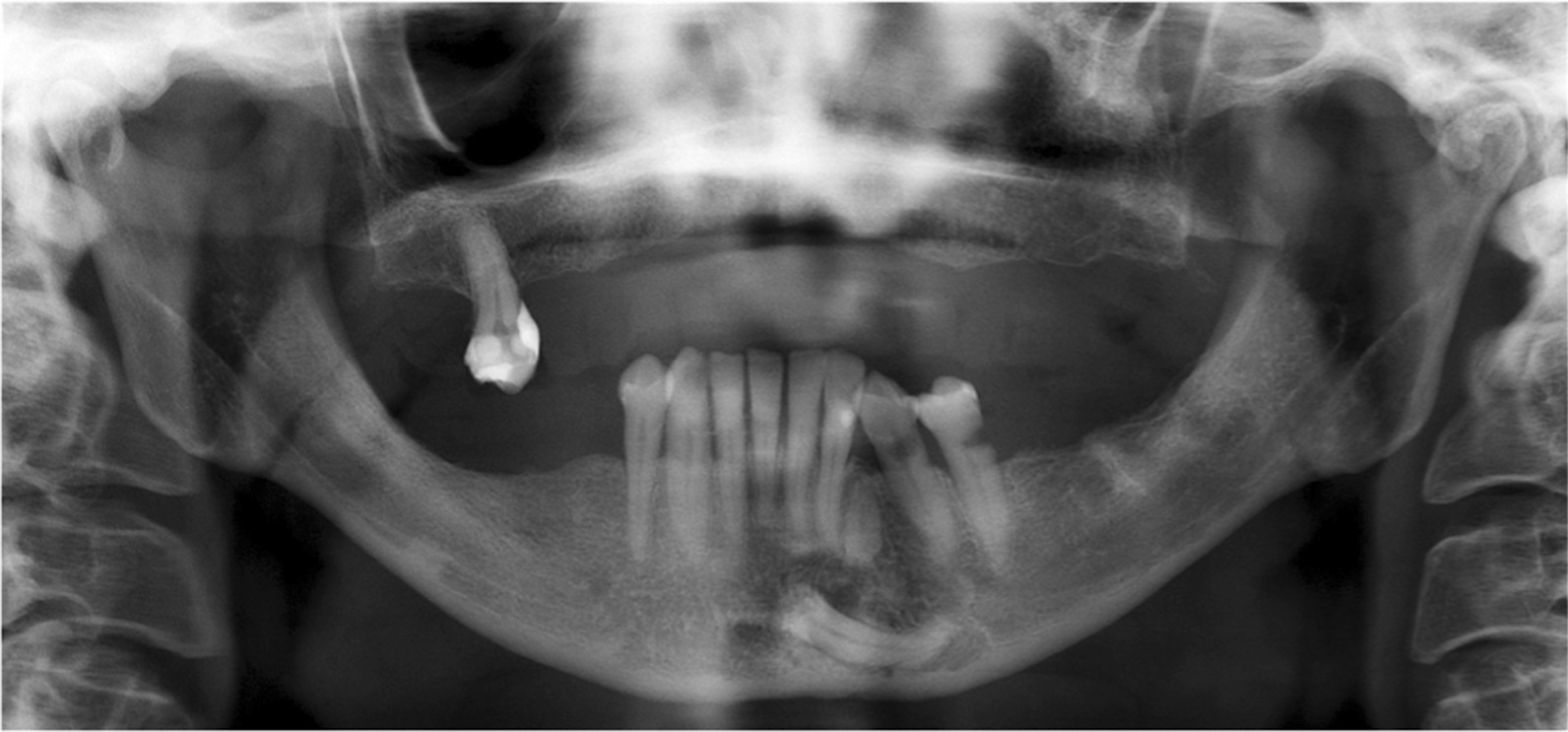
Case 1: Initial orthopantomogram revealing a round, well-circumscribed radiolucency around the upper cervical margin of the impacted left mandibular canine #33

Panoramic radiography revealed a horizontally and deeply impacted lower left canine (tooth #33) with a small, round, well-circumscribed radiolucency on its upper cervical margin. We were also able to visualize the remaining intra-osseous root of the deciduous lower left canine (tooth #73) between the roots of teeth #32 and #34 (Fig. 1).

**Fig. 2:**
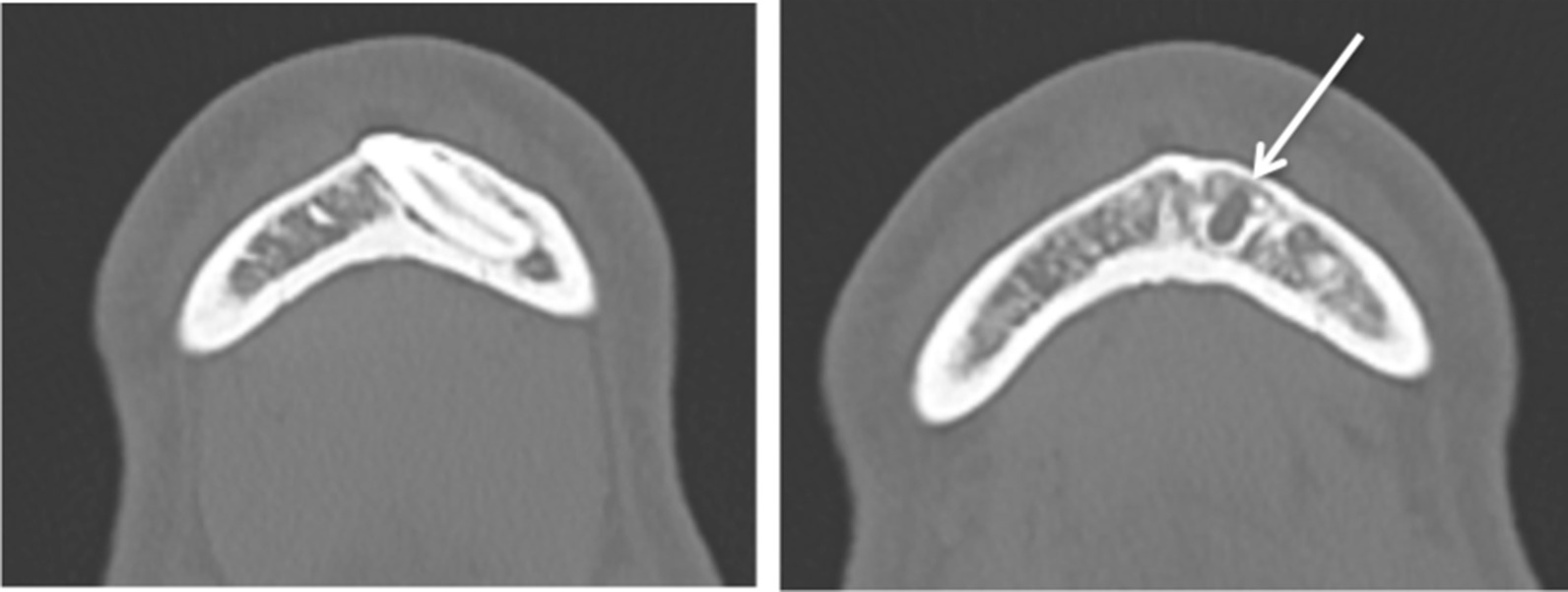
Case 1: Cone Beam CT, horizontal sections confirming impacted canine #33 (right), and the upper pericoronal radiolucency of 5 mm at its widest diameter around the collar (left)

A CT scan revealed a pericoronal radiolucency of 5 mm at its widest diameter around the impacted tooth #33 compatible with a dentigerous cyst. The impacted tooth #33 was located left of the mental foramen (Fig. 2).

In order to remove all of the oral potential infectious foci, teeth #33, #34, #35, and #73 were extracted under local anesthesia.

**Fig. 3:**
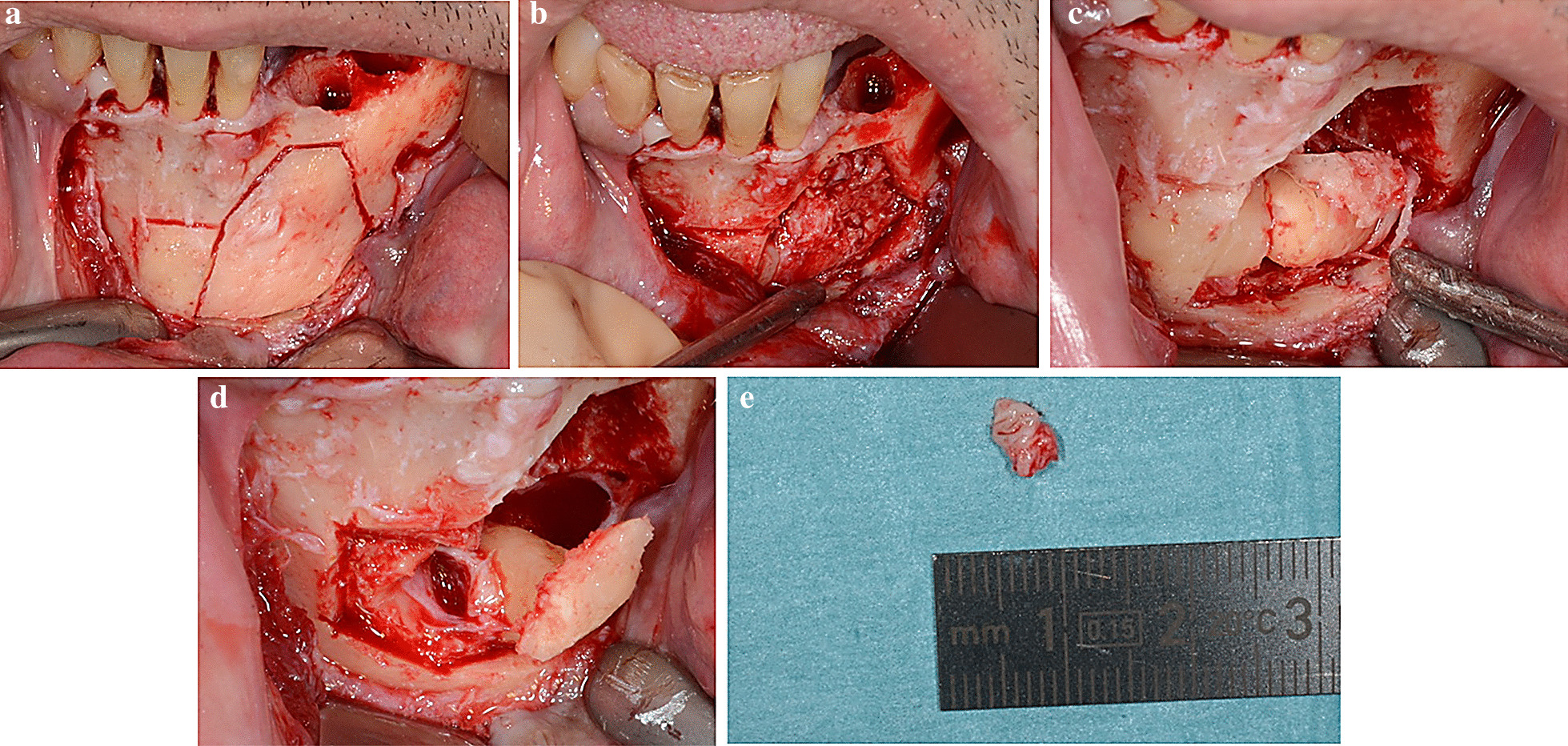
Case 1: Surgical procedure for case 1. Enucleation of the LPC and extraction of the associated impacted tooth #33. Note the bone lid that was generated by piezosurgery (**a**) and then retracted to expose tooth #33 (**b**), which was removed as two fragments (**c**, **d**) after enucleation of the LPC (**e**) on its upper cervical margin

A vestibular and lingual local anesthesia of the area of teeth #41 to #36 was carried out with a solution of articaine 4% and adrenaline 1/200,000. Simple luxation and avulsion of teeth #34 and #35 was carried out. A crestal and intra-sulcular incision from the sites of teeth #35 to #41 was made with an additional vestibular incision in the region of tooth #41. A vestibular flap was raised. The mental nerve was exposed and gently secured. A bone lid was generated by piezosurgery and then retracted to expose teeth #33 and #73. Removal of tooth #73 was carried out by periradicular osteotomy and tooth #33 was removed after corono-radicular separation. The pericoronal tissue around tooth #33 had a pearly white appearance and it was removed and sent for histopathology analysis. A 0.5% saline solution was applied, and the bone lid was replaced in its original position with platelet-rich fibrin (PRF) membranes. The surgical site was sutured and the hemostasis was assessed (Fig. 3).

One gram of co-amoxicillin was used twice a day for 1 week as prophylaxis, and 600 mg of ibuprofen twice a day and 1 g of acetaminophen three times a day were prescribed in order to control the pain. In addition, 40 mg of prednisone was prescribed for 3 days.

**Fig. 4:**
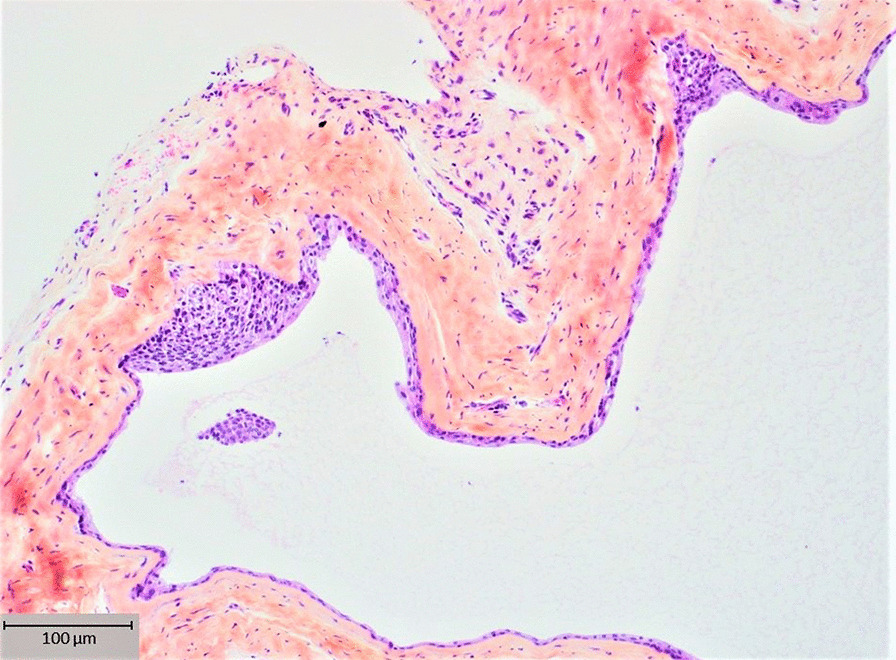
Case 1: Histological view of the LPC with a typical epithelial plaque seen by hematoxylin, eosin, and safranin staining. Unicystic cavity lined by one to five thin layers of a cuboidal and stratified non-keratinizing squamous epithelial cells, supported by a connective tissue wall devoid of inflammatory cells, containing clear cells and epithelial rests of Malassez

The histological diagnosis of the pericoronal tissue around the impacted tooth #33 revealed an LPC (Fig. 4).

The patient was seen for a check-up once a week for 1 month and the sutures were removed 2 weeks after the procedure. The postoperative course was uneventful and gingival epithelialization was obtained after 15 days.

A clinical and radiographic follow-up at 6 months and at one year revealed good healing and no complications or recurrence.

### Case 2

**Fig. 5:**
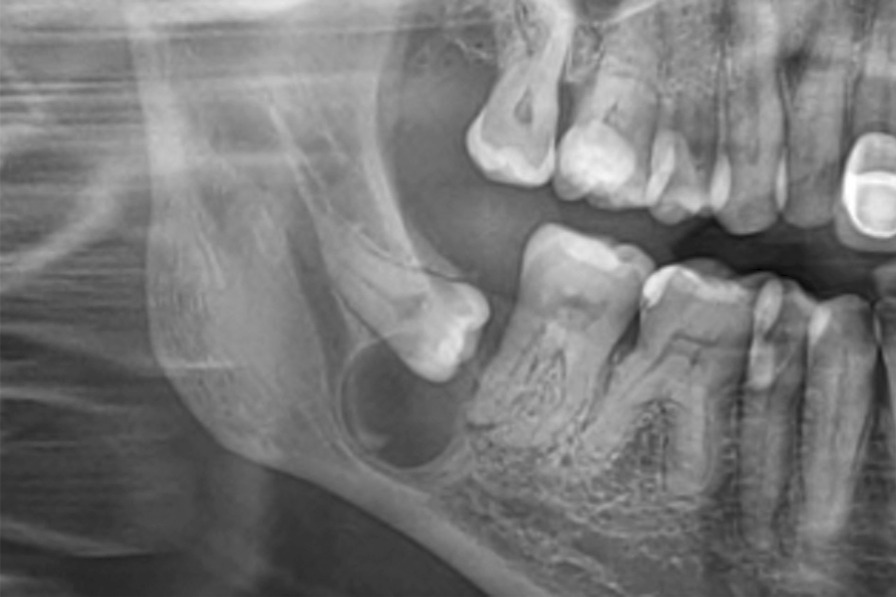
Case 2: Initial orthopantomogram revealing a round, well-circumscribed radiolucency with a diameter of about 1 cm around the lower part of the crown of the impacted horizontal tooth #48

**Fig. 6:**
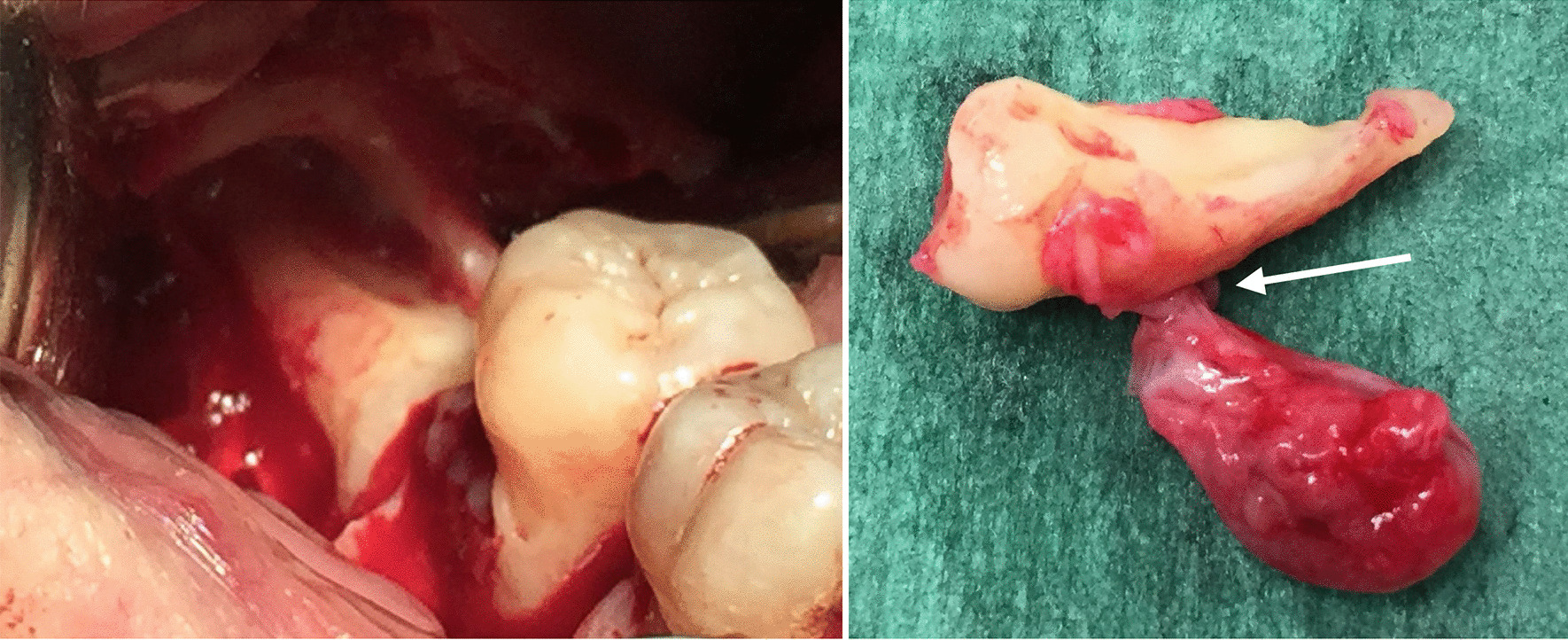
Case 2: Surgical view (on the right) of the impacted horizontal tooth #48 and gross view (on the left) of the cyst which was attached (white arrow) to the lower surface of the root of tooth #48

**Fig. 7:**
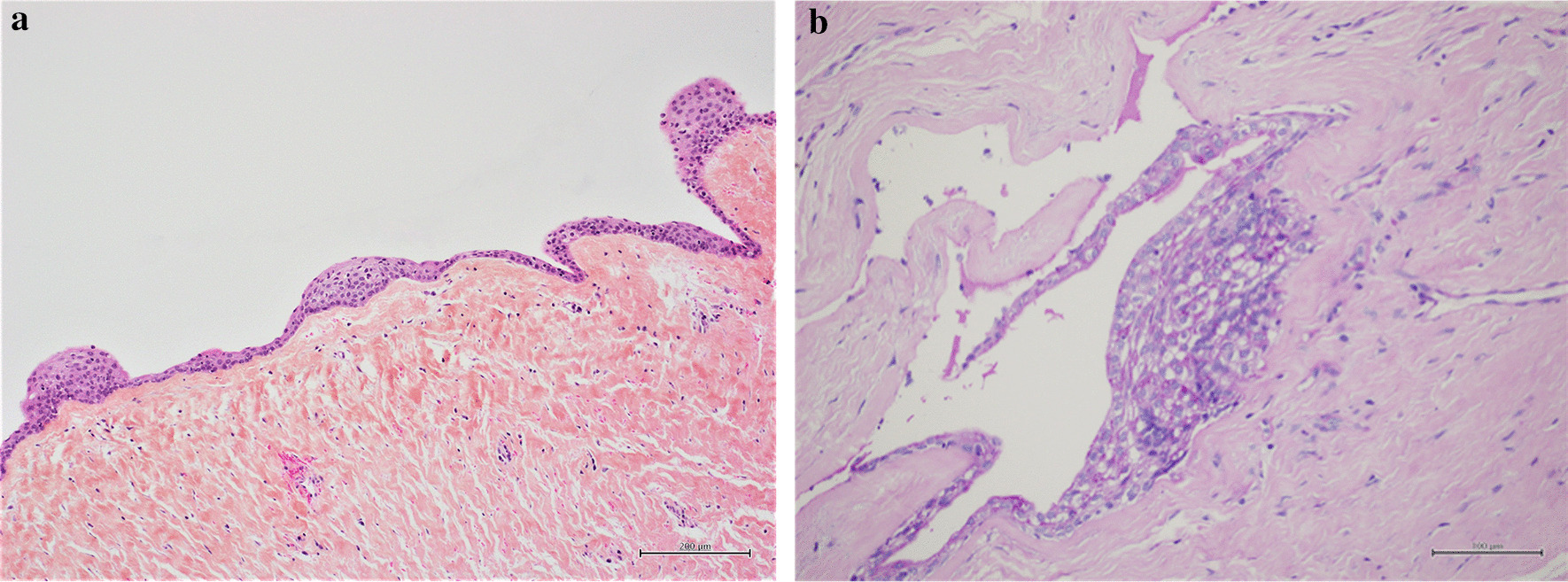
Case 2: Histological view (**a**) of the LPC with a typical epithelial plaque seen by hematoxylin, eosin, and safranin staining similar to case 1, and a high-power view (**b**) revealing PAS positive staining of clear cells in an epithelial plaque

A 54-year-old woman with healthy medical history, consulted with the main issue being pain on the lower right side of the face. Intra-oral examination revealed a vestibular swelling involving the region of the second molar (tooth #47), with obliteration of the vestibule. Distal and vestibular periodontal probing revealed a pocket depth of 9 mm and pus exuding from the gingival sulcus. A diagnosis of gingival abscess resulting from chronic periodontitis was made. Radiography also revealed a radiolucent cystic lesion with a diameter of approximately one centimeter, associated with an impacted horizontal lower right third molar (tooth #48), suggestive of a dentigerous cyst. (Fig. 5) Panoramic radiography also revealed alveolar bone resorption on the molar (tooth #47). Supragingival irrigation with chlorhexidine was carried out and antibiotics were prescribed (amoxicillin 1000 mg) along with analgesics (ibuprofen 600 mg), and the patient was instructed to rinse twice daily with 0.12% chlorhexidine. Seven days later, the cyst was removed along with the third molar and submitted for histopathological analysis, which confirmed a diagnosis of LPC with similar characteristics as case 1 (Figs. 6 and 7). The healing was uneventful. Follow-up at 18 months indicated no recurrence of the lesion.

## Discussion and conclusions

Lateral periodontal cyst (LPC) is a relatively rare developmental odontogenic cyst of the jaws. It is considered to be the intraosseous counterpart of gingival cyst in adults [4], which represents the main differential diagnosis of LPC as it also occurs in the same region, has the same epidemiology, although it predominates slightly in women, and it shares the same histopathological features [1, 4]. The difference can be assessed by its extra-osseous localization, unlike LPC, which develops inside the alveolar bone.

As the name suggests, LPCs are usually located in the interradicular region between two erupted teeth or in the alveolar bone lateral to an erupted tooth. Most cases arise in the premolar and canine areas and have a diameter of less than one centimeter. Aberrant cases of LPC have also been found in periapical sites [9, 10], very rarely and only one case in association with an unerupted tooth [11], and with a diameter in excess of a centimeter [12]. Some authors have described a small number of cases involving multifocal LPC [3].

Differential diagnosis of LPC includes various challenging radiolucent lesions because either the non-specific clinical presentation or because the lesion may be detected incidentally. Among the most common lesions that need to be considered are:

*Dentigerous cysts (DCs)*, also called follicular cysts, which are most often present as a well-defined unilocular radiolucency on radiography and they often have a sclerotic rim, usually around the crown of an impacted inferior third molar or maxillary canine [13]. Three different radiographic relationships between the tooth involved and the cyst have been described. The most common is the central variety, where a cyst develops around the tooth crown and then completely surrounds it. This cystic lesion appears to be attached to the tooth’s neck. With the lateral variety, the cyst develops around the lateral part of the tooth root and it only partially surrounds the crown. The circumferential cyst variety develops around the crown and extends down the root(s), thus the roots also appear within the cyst. Case 1 did not exhibit these imaging features while case 2 was compatible with the first variety. Moreover, the DC wall has a thin epithelial lining that usually consists of only two to three layers of cuboidal cells. The presence of focal and plaque-like thickenings permitted differential diagnosis in case 2 between LPC and DC [13].

*Radicular cysts* are usually located in the apical region of an infected non-vital tooth, although they can also develop on the lateral root surface when they are caused by an infected lateral root canal. They can usually be readily excluded due to their inflammatory characteristics and their association with a non-vital tooth.

*Glandular odontogenic cysts (GOC) or sialo-odontogenic cysts* are a type of rare developmental odontogenic cyst located intra-osseous in tooth-bearing areas. In contrast to LPCs, GOCs can exhibit aggressive behavior, with a high risk of cortical perforation and recurrence [14]. They can be distinguished from LPCs by histological analysis as GOCs contain intraepithelial microcysts, crypts, or duct-like spaces lined by a single layer of cuboidal to columnar cells with mucous cells and usually ciliated cells, which are not present in LPCs [3, 14].

Other potential differential diagnoses such as keratocyst, unicystic ameloblastoma, paradental cysts, traumatic bone cyst, adenomatoid odontogenic tumor, and giant-cell granuloma should be excluded if the clinical and histological features of an LPC are not met.

The exact origin of LPCs is still controversial and many hypotheses have been presented in the literature. Some authors suggest that LPCs are derived from epithelial remnants of the *dental lamina*, also called the glands of Serres [5]. This theory is supported by the location of LPCs, which arise along the facial portion of the alveolar process, which contain a high level of these dental lamina remnants [2]. Moreover, the same authors mention a possible related explanation for the development of BOCs, which they claim may be linked to cystic degeneration and subsequent fusion of several clusters of these dental lamina remnants [2].

Another hypothesis involves desquamation of some lateral parts of the *reduced enamel epithelium*, originally covering the crown of an unerupted tooth, in the apical direction prior to the eruption of the dental crown [5]. This assumption is supported by the resemblance between the epithelial lining of the LPC and the reduced enamel epithelium [3]. However, the late development of LPCs would imply a latent stage of these epithelia for many years before the emergence of the cyst [4].

Another hypothesis involves epithelial *rests of Malassez* left in the periodontal ligament by the breakdown of the epithelial root sheath of Hertwig [3, 5]. Buckley et al*.* support this theory, with the description of a case in which an LPC and a dentigerous cyst were associated with an unerupted lower third molar tooth [15].

Here we report two cases of unusual localization of LPC, as they were related to the pericoronal area of two impacted teeth. Our findings appear to support the theory that LPCs might arise from the epithelium rest of Malassez in the periodontal ligament, as proposed by Buckley et al*.* in their case report in 1989 [15].

These cases also underline the importance of the histopathological analysis in reaching a valid diagnosis, as the clinical and radiological features are not systematically taken into account, especially for LPC as it is a rare lesion for which the etiopathogenesis has remained controversial. To support the considerable relevance of histological analysis, the WHO has changed the LPC from a clinico-radiological entity to a histopathological one [5]. Presently, the diagnosis of LPC, therefore, appears to be primarily based on histopathologic features.

Surgical enucleation of LPC with extraction of the impacted tooth in the same procedure is the most appropriate treatment and it very rarely results in a recurrence [1]. When an LPC is associated with an erupted vital tooth, a conservative approach is required. Some authors have proposed combining laser therapy or guided bone regeneration to improve bone healing [16, 17]. In our two cases, a conventional surgical approach with appropriate curettage without bone filling resulted in good healing with no signs of recurrence at more than one year of follow-up.

In conclusion, LPC is an uncommon intra-osseous cyst that is predominantly located in the interradicular space between mandibular vital premolars. However, it can also occur less frequently at other sites, as we presented a case of an LPC that developed in the pericoronal area of an unerupted mandibular canine. Therefore, as supported by the WHO, it would be preferable to primarily define LPC based on its specific histological features and secondarily on its clinical, radiological, and epidemiological characteristics, which are less consistent.

## Data Availability

Not applicable.

## Abbreviations

LPC: Lateral periodontal cyst
BOC: Botryoid odontogenic cyst
GOC: Glandular odontogenic cyst
DC: Dentigerous cyst
